# The Metabolites of the Dietary Flavonoid Quercetin Possess Potent Antithrombotic Activity, and Interact with Aspirin to Enhance Antiplatelet Effects

**DOI:** 10.1055/s-0039-1694028

**Published:** 2019-07-30

**Authors:** Alexander R. Stainer, Parvathy Sasikumar, Alexander P. Bye, Amanda J. Unsworth, Lisa M. Holbrook, Marcus Tindall, Julie A. Lovegrove, Jonathan M. Gibbins

**Affiliations:** 1 Institute for Cardiovascular and Metabolic Research, School of Biological Sciences, University of Reading, Reading, United Kingdom; 2 Centre for Haematology, Imperial College London, London, United Kingdom; 3 School of Healthcare Science, Manchester Metropolitan University, Manchester, United Kingdom; 4 School of Cardiovascular Medicine and Sciences, King's College London, London, United Kingdom; 5 Department of Mathematics and Statistics, University of Reading, Reading, United Kingdom; 6 Department of Food and Nutritional Sciences, Hugh Sinclair Unit of Human Nutrition, University of Reading, Reading, United Kingdom

**Keywords:** platelets, quercetin, aspirin, flavonoids, thrombosis

## Abstract

Quercetin, a dietary flavonoid, has been reported to possess antiplatelet activity. However, its extensive metabolism following ingestion has resulted in difficulty elucidating precise mechanisms of action. In this study, we aimed to characterize the antiplatelet mechanisms of two methylated metabolites of quercetin—isorhamnetin and tamarixetin—and explore potential interactions with aspirin. Isorhamnetin and tamarixetin inhibited human platelet aggregation, and suppressed activatory processes including granule secretion, integrin αIIbβ3 function, calcium mobilization, and spleen tyrosine kinase (Syk)/linker for activation of T cells (LAT) phosphorylation downstream of glycoprotein VI with similar potency to quercetin. All three flavonoids attenuated thrombus formation in an in vitro microfluidic model, and isoquercetin, a 3-O-glucoside of quercetin, inhibited thrombosis in a murine laser injury model. Isorhamnetin, tamarixetin, and quercetin enhanced the antiplatelet effects of aspirin more-than-additively in a plate-based aggregometry assay, reducing aspirin IC _50_ values by an order of magnitude, with this synergy maintained in a whole blood test of platelet function. Our data provide mechanistic evidence for the antiplatelet activity of two quercetin metabolites, isorhamnetin and tamarixetin, and suggest a potential antithrombotic role for these flavonoids. In combination with their interactions with aspirin, this may represent a novel avenue of investigation for the development of new antithrombotic strategies and management of current therapies.

## Introduction

Platelets are small, anucleate cells critical to the hemostatic process. 1 Upon vessel injury, platelets are activated via interaction of cell surface receptors with collagen and subsequent activation via soluble agonists such as adenosine diphosphate (ADP), thromboxane A2 (TXA2), and thrombin, leading to a cascade of intracellular events including calcium mobilization, granule secretion, and integrin activation. 2 This leads to the formation of platelet aggregates, development of thrombi, and the cessation of bleeding. The activation of this cascade under pathophysiological conditions can, however, result in the occlusion of vessels through thrombus formation, or thrombosis, leading to events such as stroke or myocardial infarction. 3 The pharmacological targeting of platelet activation pathways with antiplatelet medications has proven very successful; medications such as aspirin, the most widely used agent worldwide, are, however, associated with a lack of effect in some patients, and are associated with adverse events such as bleeding, raising a priority for the development of more efficacious and safer alternative therapeutic approaches. 4 5

The link between diet and cardiovascular disease (CVD) is well established, with many cohort studies demonstrating the beneficial effects of fruit and vegetable intake, and specifically flavonoid intake, on cardiovascular health. 6 7 Quercetin is a commonly consumed flavonoid found in foods such as apples, onions, and red wine, and has demonstrated beneficial properties in numerous studies reporting anticancer, antimicrobial, and antiviral effects. 8 9 In addition to this, quercetin intake has been implicated in reduced CVD risk in both interventional and observational studies, with intake linked to a reduction in plasma low-density lipoprotein, reduced systolic and diastolic blood pressure, and reduced risk of ischemic heart disease. 10 11 Quercetin is also reported to possess antiplatelet effects, with an inhibition of platelet aggregation upon in vitro addition as well as ex vivo post-supplementation. 12 13 These antiplatelet effects have not, however, been fully characterized.

Upon consumption, quercetin is metabolized extensively in the small intestine and liver, yielding conjugated metabolites; the antiplatelet effects of these in vivo “effectors” have received considerably less attention. 14 Further to this, the potential for interactions between quercetin, its metabolites, and currently used antiplatelet medications such as aspirin is an important avenue of investigation; interactions between these substances must be explored in order to understand the nature of antiplatelet efficacy upon their simultaneous presence in plasma.

In this study, we investigate the antiplatelet effects of isorhamnetin and tamarixetin, two methylated metabolites of quercetin, which were found to inhibit platelet function through effects on granule secretion, integrin activation, calcium mobilization, and phosphorylation of key signaling proteins downstream of glycoprotein VI (GPVI), resulting in antithrombotic effects in vitro *.* In addition, antithrombotic effects of isoquercetin were displayed in a murine model of thrombosis, and we provide compelling evidence that quercetin and its metabolites synergize with aspirin in the inhibition of platelet function.

## Materials and Methods

### Materials

Tamarixetin, isorhamnetin, and quercetin-3-glucuronide were from Extrasynthese (Lyon, France). Isoquercetin was from Quercegen Pharmaceuticals LLC (Massachusetts, United States). Fibrillar type I collagen was from Takeda (Linz, Austria), and cross-linked collagen-related peptide (CRP-XL) was provided by Professor Richard Farndale (University of Cambridge, United Kingdom). Phycoerythrin (PE)/Cy5 antihuman CD62P antibody was from BD Biosciences (New Jersey, United States), and fluorescein isothiocyanate (FITC) antihuman fibrinogen antibody was from Dako (Glostrup, Denmark). Anti-LAT (linker for activation of T cells) phosphotyrosine 200 and anti-Syk (spleen tyrosine kinase) phosphotyrosine 525 + 526 antibodies were from Abcam (Cambridge, United Kingdom). Alexa-Fluor 488 donkey antigoat, Alexa-Fluor 647 donkey antirabbit conjugated antibody, and Fura-2 AM were from Life Technologies (Paisley, United Kingdom). Anti-β3 integrin (N-20) goat polyclonal antibody was from Santa-Cruz Biotechnology (Heidelberg, Germany). DyLight 649 conjugated anti-GPIbα antibody was from EMFRET analytics (Würzburg, Germany). CHRONO-LUME ATP detection kit was from Chronolog (Pennsylvania, United States) and Alexa-Fluor 488 conjugated phalloidin was from Thermo-Fisher Scientific (Massachusetts, United States). Precision Plus Protein dual color standard, 10X Tris/Glycine/SDS buffer, Immun-Blot PVDF membrane, and 4–20% Mini-PROTEAN TGX (tris-glycine eXtended) precast protein gels were from Bio-Rad (Hertfordshire, United Kingdom). 96-well flat bottom plates were from Greiner Bio-One (Stonehouse, United Kingdom). Vena8 Fluoro+ biochips were from Cellix Ltd (Dublin, Ireland). Platelet function analyzer (PFA)-100 cartridges were from Sysmex (Milton Keynes, United Kingdom). All other reagents were from Sigma Aldrich (Gillingham, United Kingdom).

### Methods

Light transmission measured via aggregometry, P-selectin exposure and fibrinogen binding measured via flow cytometry, dense granule secretion measured via lumi-aggregometry, adhesion and spreading on fibrinogen, clot retraction, and calcium mobilization were performed using standard techniques as described previously. 15 16 17 18 19 Immunoblotting was performed using standard protocols as described previously, with proteins of interest detected using specific antibodies raised against them and fluorophore-conjugated secondary antibodies. 16 A Typhoon FLA 9500 scanner (GE Healthcare) was used to visualize proteins, and protein levels were normalized to loading controls.

### Platelet Preparation

Washed platelet preparation was performed via differential centrifugation as described previously. 16 Platelets were resuspended to the appropriate density in modified Tyrodes-HEPES buffer (134 mM NaCl, 2.9 mM KCl, 12 mM NaHCO _3_ , 0.34 mM Na _2_ HPO _4_ , 1 mM MgCl _2_ , 20 mM N-2-hydroxyethylpiperazine-N-2-ethanesulfonic acid, 5 mM glucose, pH 7.3) and rested at 30 °C for 30 minutes before use.

### Plate-Based Aggregometry

Plate-based aggregometry was performed as previously described by Chan et al. 20 Washed platelets (2 × 10 ^8^ cells/mL) or platelet-rich plasma (PRP) were loaded into 96-well plates and incubated with vehicle control or flavonoids (5 minutes in washed platelets, 30 minutes in PRP), aspirin (30 minutes), or both, prior to agonist addition, after which plates were shaken at 1,200 rpm for 5 minutes at 37 °C using a BioShake iQ (Quantifoil Instruments, Jena, Germany). Transmission of light (405 nm) was measured using a NOVOstar plate reader (BMG Labtech, Aylesbury, United Kingdom ), and values converted to percentage aggregation using unstimulated and agonist-stimulated samples to represent 0 and 100%, respectively.

### In Vitro Thrombus Formation

Citrated human whole blood was incubated with 5 μM DiOC _6_ for 1 hour at 30 °C, and Vena8 BioChip microfluidic channels were coated for 1 hour with type I collagen at room temperature; excess collagen was washed through the channels with modified Tyrodes-HEPES buffer. Whole blood was incubated with flavonoids or vehicle control for 10 minutes at 30 °C prior to perfusion through microfluidic channels at a shear rate of 20 dyn/cm ^2^ . Using a Nikon A1-R confocal microscope, fluorescence was excited at 488 nm, and emission detected at 500 to 520 nm; the channel was observed under a 20× objective and images captured every second for 600 seconds. Mean fluorescence intensity was subsequently calculated with NIS Elements software (Nikon, Tokyo, Japan).

### Thrombus Formation In Vivo

C57/BL6 mice were dosed with isoquercetin twice per day by oral gavage, once in the morning and once in the afternoon. This was performed for 48 hours prior to commencement of the experiment, with one final dose in the morning of the experiment. Mice were anaesthetized by intraperitoneal ketamine (125 mg/kg), xylazine (12.5 mg/kg), and atropine (0.25 mg/kg) injection, and maintained with pentobarbital (5 mg/kg) as required, through a carotid artery cannula. The cremaster muscle of the testicle was affixed over a glass slide, and platelets were labeled with DyLight 649 anti-GPIbα antibody (0.2 μg/g mouse weight). Arteriole walls were injured using a Micropoint ablation laser unit (Andor Technology PLC, Belfast, Northern Ireland), with thrombus formation observed using an Olympus BX61W1 microscope (Olympus Corporation, Tokyo, Japan). Images were captured prior to and after injury using a Hamamatsu digital camera (C9300 charge-coupled device camera; Hamamatsu Photonics UK Ltd, Hertfordshire, United Kingdom) in 640 × 480 format. Images were analyzed using Slidebook 6 software (Intelligent Imaging Innovations, Colorado, United States). Experiments in mice were performed under a UK Home Office license after approval from the University of Reading Local Ethical Review Panel.

### Platelet Function Analyzer 100

Citrated human whole blood was incubated with flavonoids, aspirin, or vehicle control for 30 minutes at 30 °C, after which 800 μL was pipetted into the reservoir of PFA-100 test cartridges. Cartridges were loaded into the PFA-100 analyzer carousel and samples were run in an automated process, drawing blood through an aperture in a collagen/ADP or collagen/epinephrine-coated membrane at a shear rate of 5,000 to 6,000 s ^−1^ , and outputting a closure time (CT) upon occlusion.

### Statistical Analysis

Statistical analyses were performed using Prism Version 7 (Graphpad, California, United States). Aspirin plus flavonoid aggregometry data were analyzed by two-way analysis of variance (ANOVA) with posthoc Dunnett's multiple comparisons test. In vitro thrombus formation and protein phosphorylation data were analyzed by two-way ANOVA with posthoc Bonferroni multiple comparison test. In vivo thrombus formation data were analyzed by unpaired *t* -tests, and synergistic (more-than-additive) effects were established using paired *t* -tests. All other data were analyzed by one-way ANOVA with posthoc Dunnett's multiple comparison test. Data are presented as mean ± standard error of mean (SEM), with *p* ≤ 0.05 considered statistically significant.

## Results

### Tamarixetin and Isorhamnetin Inhibit Platelet Aggregation Evoked by Collagen

Collagen is exposed on the subendothelium upon vessel injury, and is a key activatory substance in the hemostatic response. 21 We therefore investigated whether key metabolic changes to quercetin (i.e., methylation) affected the ability to inhibit collagen-stimulated washed platelet aggregation. Reported physiological plasma concentrations vary widely according to supplementation/dietary method, dose, and the form of quercetin administered; a range between 0 and 10 μM was used here (and throughout) to represent a feasible physiological concentration range (with the upper level of 10 μM representing a high yet achievable plasma concentration of total quercetin). 12 22 23

**Fig. 1:**
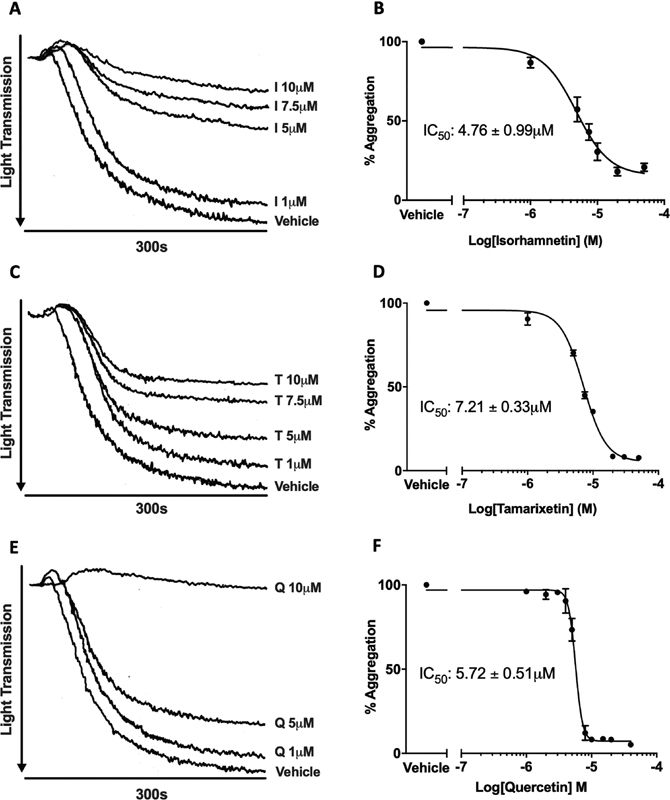
Isorhamnetin and tamarixetin, the methylated metabolites of quercetin, inhibit collagen-stimulated platelet aggregation. Washed human platelets (2 × 10 ^8^ cells/mL) were incubated with isorhamnetin ( **A, B** ), tamarixetin ( **C, D** ), quercetin ( **E, F** ), or vehicle (DMSO, 0.2% v/v) for 5 minutes (with 10 seconds stirring to mix) prior to addition of collagen (5 µg/mL). Aggregation was measured for 5 minutes at 37 °C under constant stirring (1,200 rpm) conditions in a Chrono-Log optical platelet aggregometer. ( **A, C, E** ) Representative traces from aggregation assays. ( **B, D, F** ) Four-parameter nonlinear regression curves used to estimate the IC _50_ of isorhamnetin, tamarixetin, and quercetin. *N* = 3, data represent mean ± SEM. I, isorhamnetin; T, tamarixetin; Q, quercetin.

Isorhamnetin, the 3′-methylated metabolite of quercetin, concentration-dependently inhibited collagen-stimulated platelet aggregation with an IC _50_ (half-maximal inhibitory concentration) value of 4.76 ± 0.99 μM ( Fig. 1A, B ). In contrast to this, tamarixetin, the 4′-methylated metabolite of quercetin, displayed reduced potency, inhibiting platelet aggregation fully at concentrations above 20 μM, with an IC _50_ value of 7.21 ± 0.33 μM ( Fig. 1C, D ). Quercetin inhibited aggregation at concentrations >5 μM, with an IC _50_ of 5.72 ± 0.51 μM ( Fig. 1E, F ). The structures of the flavonoids investigated are shown in Supplementary Fig. S1 . These results indicate that methylation at the 3′-position (isorhamnetin) confers a moderately higher inhibitory potency compared to methylation at the 4′-position (tamarixetin) or possession of a B ring catechol moiety (quercetin). This was confirmed upon stimulation with multiple collagen concentrations, with isorhamnetin displaying the most potent inhibitory profile ( Supplementary Fig. S2 ). In addition to this, isorhamnetin, tamarixetin, and quercetin inhibited aggregation evoked by ADP, U46619 (a stable TXA2 analog), and thrombin, providing evidence for G-protein coupled receptor (GPCR)-mediated pathway inhibition by the methylated metabolites of quercetin ( Supplementary Fig. S3 ).

### Platelet Granule Secretion is Inhibited by the Methylated Metabolites of Quercetin

**Fig. 2:**
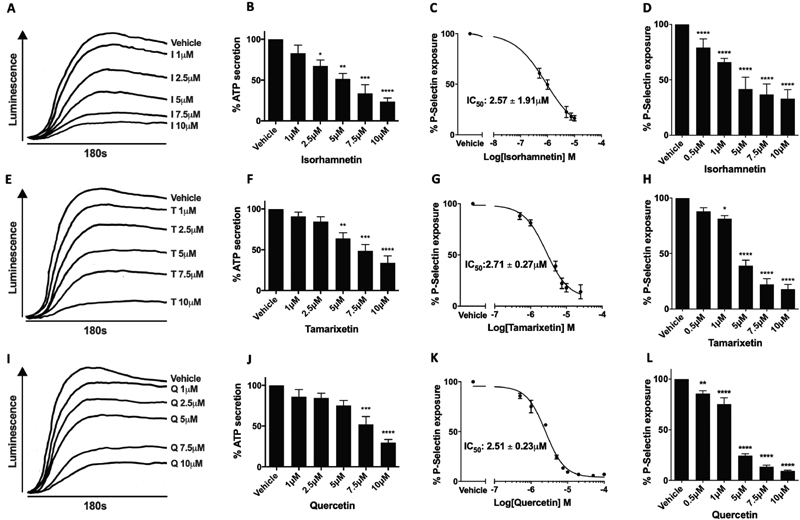
Isorhamnetin, tamarixetin, and quercetin inhibit platelet granule secretion. Washed platelets (4 × 10 ^8^ cells/mL) were treated with flavonoid or vehicle control (DMSO, 0.25% v/v) for 5 minutes, with 50 μL Chronolume luminescent substrate added in 2 minutes prior to stimulation. Collagen (5 μg/mL) was added to stimulate platelets and ATP secretion was monitored for 180 seconds under stirring conditions (1,200 rpm) at 37 °C. ( **A, E, I** ) Representative traces showing increase in luminescence upon ATP secretion and inhibition by flavonoids. ( **B, F, J** ) ATP secretion as a percentage of the amount secreted in the absence of isorhamnetin, tamarixetin, or quercetin (vehicle). Washed platelets (2 × 10 ^8^ cells/mL) were incubated with isorhamnetin ( **C, D** ), tamarixetin ( **G, H** ), quercetin ( **K, L** ), or vehicle control (DMSO, 0.25% v/v) for 5 minutes, after which PE/Cy5 antihuman CD62P antibody was added to the sample prior to stimulation. Samples were then stimulated with CRP-XL (1 μg/mL) for 20 minutes, after which they were fixed in 0.2% paraformaldehyde. P-selectin exposure on the cell surface was then measured by flow cytometry and data normalized to the level of P-selectin exposure in the absence of flavonoid (vehicle) ( **D, H, L** ). Four-parameter nonlinear regression curves were utilized to estimate the IC _50_ of isorhamnetin ( **C** ), tamarixetin ( **G** ), and quercetin ( **K** ). *N* = 3, data represent mean ± SEM. * *p* < 0.05, ** *p* < 0.005, **** *p* < 0.0001 compared to vehicle control, analyzed by one-way ANOVA with posthoc Dunnett's test. I, isorhamnetin; T, tamarixetin; Q, quercetin.

The secretion of platelet granules is a key process driving the positive feedback cycle of activation; dense granules are rich in small molecules such as adenosine triphosphate (ATP), ADP, and serotonin, and α-granules contain proteins such as fibronectin, P-selectin, and von Willebrand factor. 24 In order to elucidate whether the antiaggregatory effects of tamarixetin and isorhamnetin were due to an upstream effect on dense granule secretion, collagen-stimulated (5 μg/mL) secretion of ATP from washed platelets was investigated. Isorhamnetin, tamarixetin, and quercetin inhibited dense granule secretion; a concentration-dependent reduction in ATP secretion was observed with isorhamnetin between 2.5 and 10 μM, with 76 ± 4.4% inhibition at the highest concentration tested (10 μM; Fig. 2A, B ). Secretion was reduced significantly by tamarixetin at concentrations between 5 and 10 μM ( Fig. 2E, F ). Quercetin effected dense granule secretion similarly to tamarixetin, with a maximal inhibition of 70 ± 3.9% at 10 μM ( Fig. 2I, J ).

The ability of tamarixetin and isorhamnetin to attenuate the exposure of P-selectin upon stimulation with collagen-related peptide (1 μg/mL CRP-XL, cross-linked: a GPVI-selective ligand that is compatible with flow cytometry analysis) was also investigated, as a marker of α-granule secretion. Isorhamnetin inhibited significantly this process at all concentrations tested, with a 39 ± 4.9% reduction in P-selectin exposure upon treatment with a low flavonoid concentration of 0.5 μM (IC _50_ : 2.57 ± 1.91 μM; Fig. 2C, D ). Tamarixetin inhibited comparably, with a significant effect at 1 μM plus and an IC _50_ of 2.71 ± 0.27 μM ( Fig. 2G, H ). Quercetin also attenuated P-selectin exposure as low as 0.5 μM (14 ± 2.7% inhibition), a readily achievable physiological concentration, with an IC _50_ of 2.51 ± 0.23 μM ( Fig. 2K, L ). Overall, these data suggest that one of the key mechanisms through which isorhamnetin and tamarixetin inhibit aggregation is through an early effect on granule secretion.

### Integrin αIIbβ3 Signaling Is Modulated by the Quercetin Metabolites Tamarixetin and Isorhamnetin

**Fig. 3:**
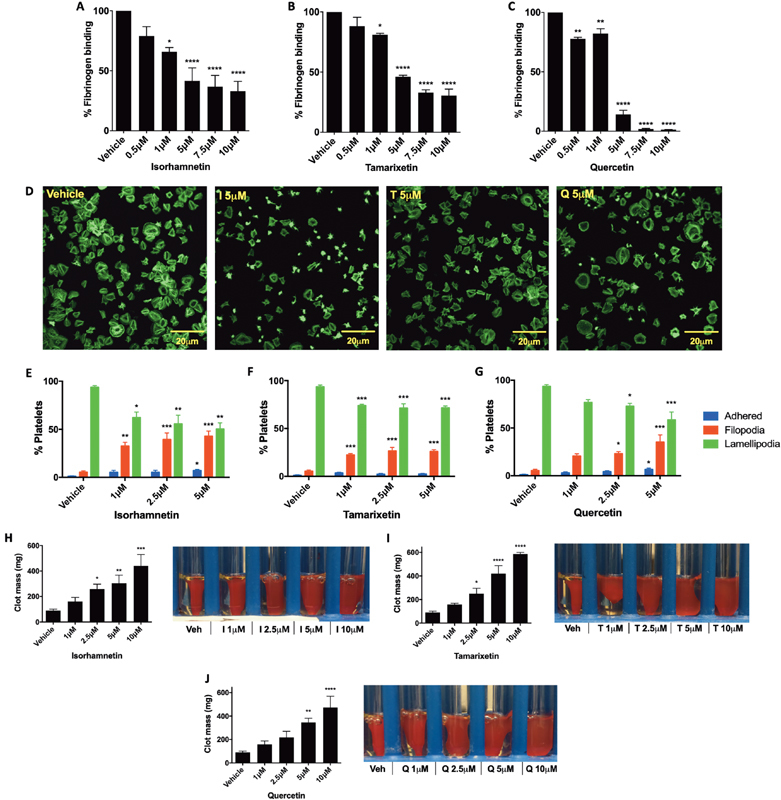
Integrin αIIbβ3 affinity and function is altered by quercetin's methylated metabolites. Washed platelets (2 × 10 ^8^ cells/mL) were incubated with isorhamnetin ( **A** ), tamarixetin ( **B** ), quercetin ( **C** ), or vehicle control (DMSO, 0.25% v/v), stimulated with CRP-XL (1 µg/mL), and fibrinogen binding measured by flow cytometry. Data normalized to the level of fibrinogen binding in the absence of flavonoid (vehicle). Washed platelets (2 × 10 ^8^ cells/mL) were incubated with isorhamnetin ( **E** ), tamarixetin ( **F** ), quercetin ( **G** ), or vehicle control (DMSO 0.33% v/v) for 5 minutes, then allowed to spread on fibrinogen (100 µg/mL) coated coverslips for 45 minutes at 37 °C. Cells were stained with Alexa fluor 488 phalloidin and visualized using a Nikon A1-R confocal microscope. Data represent percentage of platelets in each stage of spreading ( **E–G** ), mean ± SEM, with representative images in ( **D** ). PRP was incubated with isorhamnetin ( **H** ), tamarixetin ( **I** ), quercetin ( **J** ), or vehicle control (DMSO, 0.2% v/v) for 60 minutes at 30 °C, after which thrombin (1 U/mL) was added to stimulate clot formation. The assay was terminated at 120 minutes when vehicle-treated clots had retracted fully, with representative images shown as well as clot weight at 120 minutes. *N* = 4, data represent mean ± SEM. * *p* < 0.05, ** *p* < 0.005, *** *p* < 0.001, **** *p* < 0.0001 compared to vehicle control, analyzed by one-way ANOVA with posthoc Dunnett's test. I, Isorhamnetin; PRP, platelet-rich plasma; Q, quercetin; T, tamarixetin.

Upon activation, signals from within the platelet function to upregulate the affinity of integrin αIIbβ3 into an “open” state (via “inside-out signaling”), to which fibrinogen binds, ultimately leading to aggregation. 25 As it is known that quercetin functions as a broad spectrum inhibitor of protein kinases, it was hypothesized that this integrin affinity regulation may be modulated by the flavonoids. This was measured by investigating CRP-XL (1 μg/mL) stimulated fibrinogen binding to “open” integrin αIIbβ3. All three flavonoids tested inhibited significantly the binding of fibrinogen; upon treatment with isorhamnetin, a significantly increased level of inhibition at lower flavonoid concentrations (1 μM) compared to quercetin and tamarixetin was observed ( *p* < 0.05), with 34 ± 3.5% inhibition at 1 μM ( Fig. 3A ). Isorhamnetin reduced fibrinogen binding between 1 and 10 μM, as did tamarixetin, with IC _50_ values of 6.27 ± 2.81 and 5.29 ± 1.58 μM, respectively ( Fig. 3A, B ). Quercetin attenuated fibrinogen binding at all concentrations tested, with >25% inhibition at 0.5 μM. Indeed, over 95% inhibition was achieved upon treatment with 7.5 μM, with an IC _50_ value of 3.13 ± 0.46 μM ( Fig. 3C ).

Upon ligand binding to integrin αIIbβ3, receptor clustering causes “outside-in” signaling events that result in platelet spreading, platelet thrombus contraction, and ultimately downstream clot retraction. 26 In order to determine whether outside-in signaling may be affected by the methylated metabolites, platelet spreading on fibrinogen was investigated. All flavonoids tested inhibited significantly platelet adhesion on fibrinogen at 1, 2.5, and 5 μM (representative images shown in Fig. 3D ). The spreading of platelets on fibrinogen was also inhibited upon treatment with flavonoid concentrations as low as 1 μM, with fewer platelets able to fully form lamellipodia, instead progressing only to the adhered or filopodia-extending stages ( Fig. 3E–G ). Consistent with other functional data in washed platelets, isorhamnetin proved significantly more potent ( *p* < 0.05) than quercetin and tamarixetin at lower concentrations (1 μM).

Clot retraction was also investigated as a measure of outside-in signaling, utilizing PRP and a thrombin-stimulated clot. Retraction was inhibited by isorhamnetin and tamarixetin between 2.5 and 10 μM, and by quercetin between 5 and 10 μM. Indeed, Fig. 3 (H–J) shows that, upon 10 μM treatment, retraction of clots was reduced substantially after 2 hours (when vehicle-treated clots had fully retracted). Tamarixetin had the largest effect, with a 6.5× larger clot weight at 2 hours compared to vehicle control; this suggests that, in the presence of plasma proteins, a 4′-methyl group confers increased inhibitory potency. This is supported by the observation that tamarixetin inhibits significantly platelet aggregation in PRP at concentrations of 10 μM and higher, with quercetin and isorhamnetin inhibiting significantly at ≥25 μM (the higher concentrations required here may be explained by stirring conditions and use of collagen [5 μg/mL] as agonist; Supplementary Fig. S4 ). These data display inhibitory effects of quercetin and its methylated metabolites at physiologically achievable concentrations in the presence of plasma proteins, and suggest a role for these flavonoids in integrin αIIbβ3 outside-in signaling inhibition.

### Isorhamnetin Potently Inhibits CRP-XL Stimulated Cytosolic Calcium Elevation

**Fig. 4:**
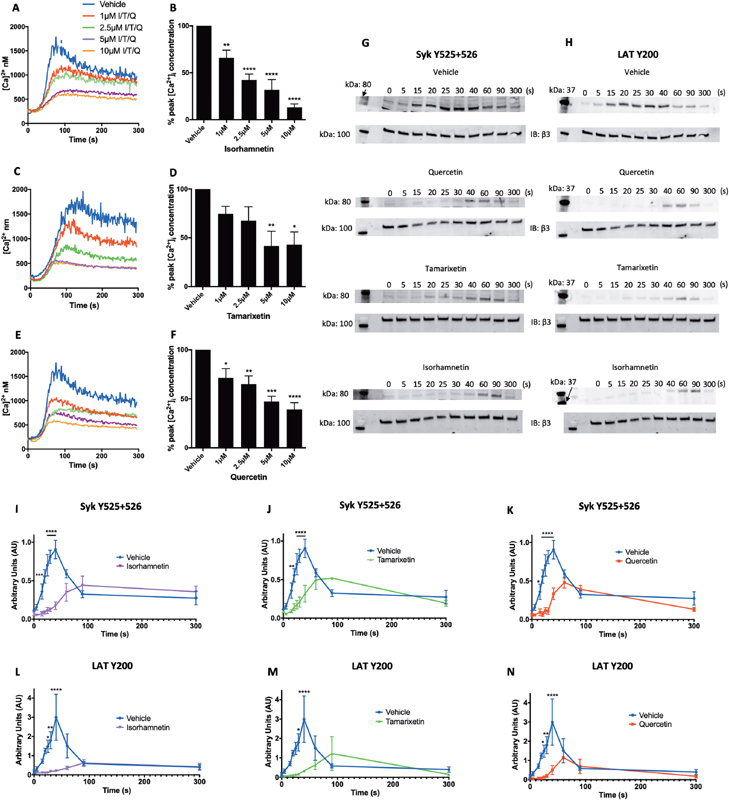
Isorhamnetin, tamarixetin, and quercetin inhibit calcium mobilization and signaling proximal to GPVI. Fura-2 loaded washed platelets (4 × 10 ^8^ cells/mL) were incubated with isorhamnetin ( **A, B** ), tamarixetin ( **C, D** ), quercetin ( **E, F** ), or vehicle control (DMSO, 0.2% v/v) for 5 minutes at 37 °C prior to stimulation with CRP-XL (1 µg/mL). Fluorescence (excitation 340 and 380 nm, emission 510 nm) was recorded for 5 minutes using a NOVOstar plate reader, and [Ca ^2+^ ] _*i*_ was estimated using the equation described in the Methods ( **A, C, E** ). Peak [Ca ^2+^ ] _*i*_ was taken as the maximum value reached in the sample over the 5-minute period, and was normalized to peak calcium levels in the absence of flavonoid (vehicle) ( **B, D, F** ). *N* = 4, data in ( **B** ), ( **D** ), and ( **F** ) represent mean ± SEM, analyzed by one-way ANOVA with posthoc Dunnett's test. * *p* < 0.05, ** *p* < 0.005, *** *p* < 0.001, **** *p* < 0.0001 compared to vehicle control. Washed human platelets (4 × 10 ^8^ cells/mL) were incubated with isorhamnetin ( **I, L** ), tamarixetin ( **J, M** ), quercetin ( **K, N** ), or vehicle control (DMSO, 0.4% v/v) for 5 minutes at 37 °C prior to addition of CRP-XL (1 μg/mL). Aggregation was allowed to occur for defined time points, after which samples were immediately lysed in 6X reducing sample buffer. Proteins were separated by SDS-PAGE and immunoblotted for phosphorylated Syk (Y525 + 526) and LAT (Y200). Levels of fluorescence of individual bands were normalized to loading controls (integrin β3), with representative blots shown in ( **G, H** ). *N* = 3, data represent mean ± SEM. * *p* < 0.05, ** *p* < 0.005, **** *p* < 0.0001 compared to vehicle control at corresponding time points, analyzed by two-way ANOVA with posthoc Bonferroni correction. I, isorhamnetin; LAT, linker for activation of T cells; Q, quercetin; SDS-PAGE, sodium dodecyl sulphate-polyacrylamide gel electrophoresis; Syk, spleen tyrosine kinase; T, tamarixetin.

The mobilization of calcium from intracellular platelet stores and the entry of calcium into the cell are crucial for platelet activation, driving granule secretion and integrin αIIbβ3 activation. 27 As these downstream processes were inhibited, it was investigated whether an upstream effect on calcium mobilization was associated with diminished platelet responses. Cytosolic calcium elevation stimulated by CRP-XL (1 μg/mL) was inhibited significantly by isorhamnetin between 1 and 10 μM, with 34 ± 8.2% inhibition at 1 μM, and a maximal effect of 87 ± 3.7% ( Fig. 4A, B ). Tamarixetin attenuated calcium elevation less potently, with a significant reduction between 5 and 10 μM ( Fig. 4C, D ). Quercetin also inhibited at all concentrations tested, with a reduced maximum effect of 61 ± 6.8% compared to isorhamnetin ( Fig. 4E, F ).

### Early Signaling Events Downstream of GPVI Are Inhibited by Isorhamnetin and Tamarixetin

GPVI is a transmembrane collagen receptor associated with an FcRγ-chain dimer. Upon ligand binding, strong platelet activation occurs, driven largely by protein kinase signaling. As the methylated metabolites of quercetin inhibited platelet activation via this pathway, their ability to inhibit phosphorylation of signaling proteins downstream of GPVI, namely Syk and LAT, was investigated. Upon stimulation with CRP-XL (1 μg/mL), Syk and LAT phosphorylation peaked at 40 seconds, decreasing after this time up to 300 seconds (representative blots are shown in Fig. 4G, H ). Upon treatment with isorhamnetin (7.5 μM, chosen as an intermediate concentration used in aggregometry assays), peak Syk phosphorylation (the highest level of phosphorylation across the time-course) was delayed to 90 seconds, and was inhibited by 51 ± 16.2% compared to control ( Fig. 4I ). Tamarixetin delayed peak Syk phosphorylation to 90 seconds, with no significant difference in peak levels ( Fig. 4J ). Quercetin treatment delayed peak Syk phosphorylation to 60 seconds, and, like isorhamnetin, inhibited it significantly (a 49 ± 12% reduction, Fig. 4K ).

Isorhamnetin potently inhibited the CRP-XL-stimulated phosphorylation of LAT, with no real peak observed and a significant reduction compared to vehicle control between 25 and 40 seconds ( Fig. 4L ). Tamarixetin again delayed peak phosphorylation up to 90 seconds, with a 90% reduction compared to vehicle control between 30 and 40 seconds ( Fig. 4M ). Quercetin treatment resulted in a delay in peak phosphorylation up to 60 seconds; between 25 and 40 seconds, significant inhibition was observed, up to 90% ( Fig. 4N ). There was, however, no effect on peak phosphorylation levels by any flavonoid, possibly due to high inter-individual variance. These data suggest that like quercetin, tamarixetin and isorhamnetin both delay and impair phosphorylation of some of the immediate effectors of the GPVI pathway, likely leading to downstream signaling alteration and the observed functional inhibition.

### Isorhamnetin and Tamarixetin Inhibit Thrombus Formation In Vitro

**Fig. 5:**
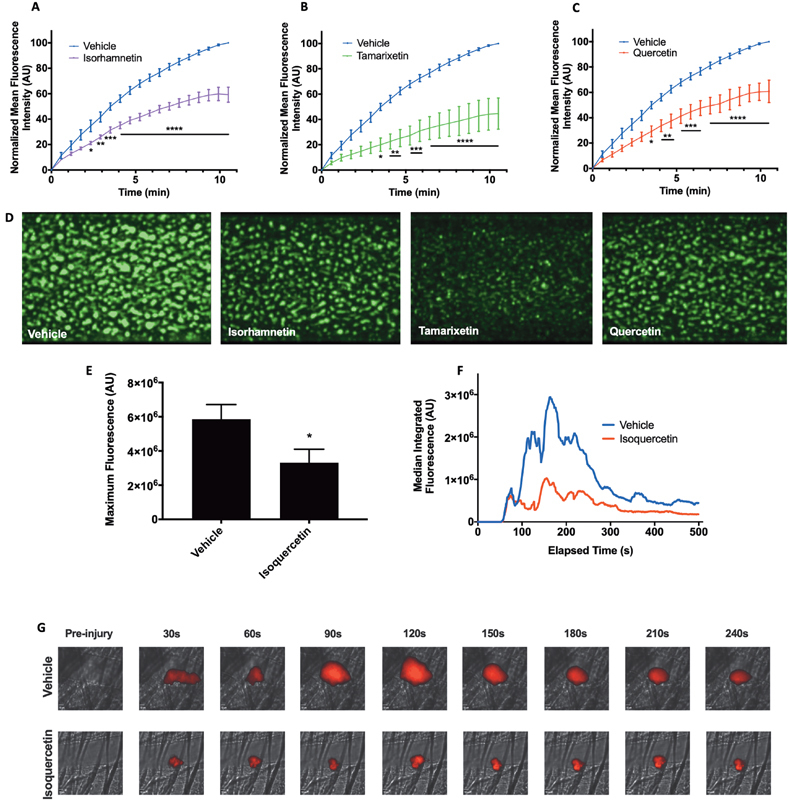
Isorhamnetin, tamarixetin, and quercetin display antithrombotic actions in vitro, and isoquercetin inhibits thrombosis in vivo *.* DiOC _6_ -labelled whole blood was incubated with isorhamnetin ( **A** ), tamarixetin ( **B** ), quercetin ( **C** ) (10 μM), or vehicle control (DMSO, 0.2% v/v) prior to perfusion through collagen-coated Vena8 biochip channels at a shear rate of 20 dyn/cm ^2^ , with images captured every second for 600 seconds. Mean fluorescence intensity was normalized to the level in the absence of flavonoid (vehicle) and was calculated using NIS Elements software ( **A–C** ), and representative images at the assay endpoint are shown in ( **D** ). C57BL/6 mice were dosed twice per day (9 am and 5 pm) with isoquercetin (200 mg/kg) or vehicle control (distilled water) by gavage for 48 hours prior to the experiment, with one final dose administered in the morning of the experiment. Mice were anaesthetized, platelets were labeled with DyLight 649 anti-GPIbα antibody, and laser injury of a cremaster muscle arteriole wall was performed with a Micropoint ablation laser unit. Thrombus formation was observed using an Olympus BX61W1 microscope, with images analyzed to calculate fluorescence intensity of thrombi using Slidebook software (version 6). Maximum fluorescence of samples reached over the assay ( **E** ). Median-integrated fluorescence values from all thrombi are shown in ( **F** ). Representative images from experiments at different time points are shown in ( **G** ); scale bars in bottom left of the images represent 10 µm. For in vitro data, *N* = 3, data represent mean ± SEM. * *p* < 0.05, ** *p* < 0.005, *** *p* < 0.001, **** *p* < 0.0001 compared to vehicle control, analyzed by two-way ANOVA with posthoc Bonferroni correction. For in vivo data, *N* = 4, with multiple thrombi per mouse. * *p* < 0.05 compared to vehicle control; data in ( **E** ) represent mean ± SEM, analyzed by unpaired *t* -test.

Due to the potent inhibitory effects of isorhamnetin and tamarixetin on platelet function, it was hypothesized that these metabolites of quercetin may demonstrate antithrombotic properties. The effects of these flavonoids on thrombus formation were therefore tested in vitro under physiological arterial flow conditions. Vehicle-treated blood perfused over collagen formed thrombi normally, increasing in size as platelets accumulated over the 10-minute assay period. Upon flavonoid treatment, thrombus formation (measured as an increase in fluorescence intensity) was inhibited from early time points, reaching significance from 140 seconds onward upon isorhamnetin treatment, and from 210 seconds onward upon tamarixetin and quercetin treatment ( Fig. 5A–C , representative images shown in D). End point fluorescence values demonstrate a 41 ± 5.9% inhibition of total thrombus formation upon isorhamnetin treatment, a 55 ± 12% reduction with tamarixetin, and 39 ± 8.9% inhibition with quercetin. This trend toward an increased effect of tamarixetin is consistent with clot retraction data in that, in the presence of plasma proteins, a 4′-methyl group may confer higher inhibitory potency over a 3′-methyl group or a B ring catechol moiety.

### Isoquercetin Inhibits Thrombus Formation in a Murine Model of Arterial Thrombosis

Having displayed high inhibitory potency in vitro, the potential effect of quercetin on thrombosis in vivo was investigated. This was achieved using a laser injury model, in which thrombi are induced in cremaster muscle arterioles and observed via intravital microscopy. Isoquercetin was used in these experiments, as the route of administration was oral, and the 3-glucoside moiety results in higher bioavailability and plasma quercetin concentrations compared to quercetin aglycone. 28 29 Indeed, studies have demonstrated isorhamnetin and tamarixetin in plasma following isoquercetin administration, and after supplementation with other quercetin glucosides. 12 30 A 48-hour dosing regimen was utilized, with morning and afternoon dosing, to mimic human supplemental consumption, and to investigate if repeated supplementation resulted in antithrombotic effects.

Treatment with isoquercetin over 48 hours resulted in a 45 ± 11.9% reduction in maximum thrombus size ( Fig. 5E ). Kinetically, upon injury, thrombi in vehicle-treated mice increased in size, reaching a peak at approximately 160 seconds, after which the thrombus size gradually declined. Upon isoquercetin administration, fluorescence levels initially rose similarly as platelets accumulated in the thrombus; however, the growth was not sustained, with thrombi becoming unstable, platelets embolizing, and thrombi unable to grow to the same degree (see Supplementary Videos 1 and 2 , and Fig. 5F ). Representative images are shown in Fig. 5G . Together, these data demonstrate the ability of an oral isoquercetin dose to inhibit thrombosis in vivo, suggesting that the antiplatelet effects of quercetin's metabolites are maintained in a whole, physiological system.

**Video 1**

Example of thrombus formation in vivo in vehicle-treated mice.

**Video 2**

Example of thrombus formation in vivo in isoquercetin-treated mice.

### Quercetin and Its Methylated Metabolites Enhance the Antiplatelet Effect of Aspirin

Aspirin use is associated with adverse bleeding events, and as such the lowering of doses may be desirable; one way to achieve this could be co-administration of flavonoid supplements. 31 32 The effects of aspirin and flavonoids on collagen (5 μg/mL) stimulated platelet aggregation, both individually and in combination, were therefore investigated.

**Fig. 6:**
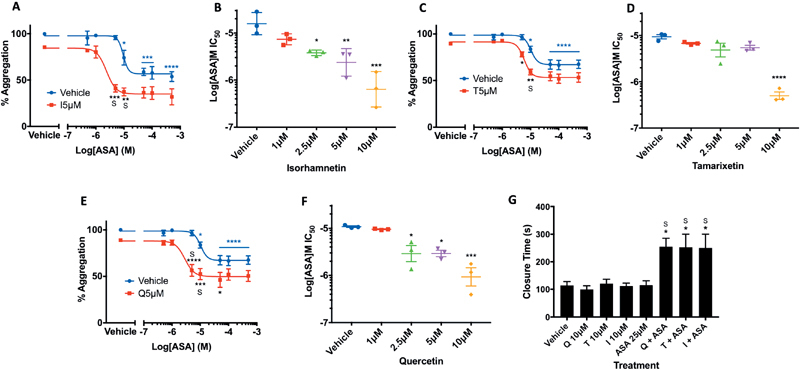
Isorhamnetin, tamarixetin, and quercetin synergize with aspirin to enhance antiplatelet effects. Washed platelets (2 × 10 ^8^ cells/mL) were incubated in a 96-well plate with aspirin ± isorhamnetin ( **A, B** ), tamarixetin ( **C, D** ), quercetin ( **E, F** ) (5 µM flavonoid shown as example), or vehicle control (0.25% ethanol + 0.5% DMSO, v/v), and aggregation stimulated by collagen (5 µg/mL). Data represent percentage aggregation normalized to levels of aggregation in the absence of flavonoid (vehicle) ( **A, C, E** ). ( **B, D, F** ) IC _50_ values for aspirin upon addition of flavonoid. *N* = 3, data represent mean ± SEM. * *p* < 0.05, ** *p* < 0.005, *** *p* < 0.001, **** *p* < 0.0001. *Blue stars* indicate a significant effect of aspirin compared to vehicle control, analyzed by one-way ANOVA. *Black stars* indicate a significant effect of flavonoid plus aspirin compared to aspirin alone, analyzed by two-way ANOVA. “S” indicates an enhanced effect of dual treatment compared to the combined effects of individual flavonoid and aspirin concentrations ( *p* < 0.05), analyzed by paired *t* -test. Whole blood was incubated with quercetin ± aspirin, tamarixetin ± aspirin, isorhamnetin ± aspirin, aspirin alone, or vehicle control (0.1% DMSO + 0.1% ethanol) for 30 minutes at 30 °C. Whole blood was loaded into the reservoir of CADP cartridges and samples were run through the PFA-100, with closure time (s) shown in ( **G** ). *N* = 3, data represent mean ± SEM. *Stars* represent a significant prolongation of closure time compared to vehicle control. * *p* < 0.05, analyzed by one-way ANOVA. “S” represents a synergistic effect as described above. ASA, acetylsalicylic acid (aspirin); CADP, collagen plus adenosine diphosphate; I, isorhamnetin; Q, quercetin; T, tamarixetin.

Aspirin alone inhibited aggregation of washed platelets at 10 μM and above; combined flavonoid and aspirin treatment resulted in a significantly higher level of inhibition compared to aspirin alone at 2.5, 5, and 10 μM isorhamnetin. A synergistic (more-than-additive) enhancement of antiplatelet effect was observed at 2.5 and 5 μM isorhamnetin; for example, treatment with 5 μM isorhamnetin and 5 μM aspirin resulted in a 59 ± 3.9% inhibition of aggregation, more than the sum of the effects of these individual concentrations (14 ± 1.4 and 4 ± 2.3% inhibition, respectively; *p* < 0.05; Fig. 6A ). In Fig. 6 , an “S” denotes a statistically significantly greater effect of dual treatment compared to the individual effects of flavonoids and aspirin added together (as opposed to simply an additive effect of including another inhibitor of platelet function). Similar results were observed for both tamarixetin/quercetin and aspirin dual treatment, with synergistic effects observed upon treatment with 2.5 and 5 μM flavonoid ( Fig. 6C, E ). The IC _50_ values for the inhibition of platelet aggregation by aspirin upon flavonoid co-administration highlight this interactive effect, with the IC _50_ value of 10.83 μM (±0.45 μM) for aspirin alone decreasing to 3.32 μM (±1.08 μM), 2.99 μM (±0.44 μM), and 1.11 μM (±0.4 μM) upon addition of 2.5, 5, and 10 μM quercetin, respectively ( Fig. 6F ). Indeed, upon co-incubation of 10 μM isorhamnetin and tamarixetin, half-maximal inhibition of aggregation was achieved at 0.8 μM (±0.36 μM) and 0.52 μM (±0.1 μM) aspirin respectively, lowering the IC _50_ value of aspirin by over an order of magnitude ( Fig. 6B, D ). Together, these data demonstrate the ability of isorhamnetin, tamarixetin, and quercetin to reduce the IC _50_ value of aspirin in the inhibition of aggregation at concentrations as low as 2.5 μM; plasma concentrations proven to be physiologically relevant. 23 33

### The Antiplatelet Interaction between Flavonoids and Aspirin is Maintained in a Whole Blood Assay of Platelet Function

Having established synergy between aspirin and the methylated metabolites of quercetin, we asked whether these interactions were maintained in a whole blood system, with the presence of interfering factors such as plasma proteins. This was tested using a PFA-100 instrument, first described by Kundu et al, which simulates primary hemostasis by drawing anticoagulated blood through an aperture cut into a membrane coated with collagen plus ADP (CADP) and providing a measurement of CT of the aperture upon its occlusion. 34 Treatment of whole blood with aspirin (25 μM), isorhamnetin, tamarixetin, or quercetin (10 μM) individually did not significantly prolong the CT. However, dual treatment with isorhamnetin and aspirin resulted in a significant prolongation of CT, from 115 seconds (±13.8) in vehicle-treated whole blood to 250 ± 50 seconds upon isorhamnetin/aspirin combined treatment. This effect was more than additive when compared to individual treatment with isorhamnetin (CT: 113 ± 9.8 s) and aspirin (CT: 115 ± 15.9 s) alone. This more-than-additive enhancement of the effect was also seen upon dual treatment with tamarixetin/aspirin and quercetin/aspirin, with average CTs of 253 ± 47 and 255 ± 30.3 seconds, respectively (tamarixetin alone, CT: 121 ± 15.9 s; quercetin alone, CT 100 ± 12.6; Fig. 6G ). These results provide evidence for the maintenance of a more-than-additive interaction between quercetin and its methylated metabolites and aspirin in the inhibition of platelet function in whole blood, highlighting a potential significance of this interaction in vivo.

## Discussion

It has been demonstrated in many studies that diets high in flavonoids are associated with reduced CVD risk, and a better understanding of the effects of dietary flavonoids on platelet function will help elucidate the mechanisms underlying this. 6 Quercetin, one of the key dietary flavonoids worldwide, has been shown to dampen platelet function; however, on ingestion it is metabolized extensively, and significantly less attention has been focused on these metabolites, the potential in vivo “effectors.” 13 35 In this study, the antithrombotic effects and underlying mechanisms of two common physiological methylated metabolites of quercetin—isorhamnetin and tamarixetin—were studied.

We report that the methylated metabolites of quercetin, isorhamnetin and tamarixetin, potently inhibit platelet aggregation stimulated by collagen, with effects observed as low as 1 μM; a total plasma quercetin concentration achievable through both diet and supplemental means. 23 33 As the primary, initial stimulus upon vessel injury, a strong inhibition of collagen-stimulated platelet aggregation by the methylated metabolites of quercetin suggests early inhibitory effects, upon initial hemostatic challenge. 21 Platelet aggregation stimulated via GPCR pathways, crucial for the positive feedback associated with platelet activation, was also affected. The ability of these flavonoids to inhibit this multitude of activatory pathways is likely through inhibition of kinases which are involved in both the initiation and propagation of platelet responses. Indeed, it has been shown that quercetin is able to inhibit the activity of many tyrosine and lipid kinases involved in platelet activation including Lyn, Fyn, Src, AKT, Syk, and phosphoinositide 3-kinase (PI3K). 13 36 The inhibition of activation through multiple GPCR pathways likely, at least in part, underlies the potent inhibition seen with these flavonoids. Secondary platelet activation through GPCR-activating ligands is key for the positive feedback mechanisms that are necessary for a rapid and robust platelet response. 2 Dampened initial responses to collagen and subsequent inhibited GPCR signaling may be responsible for the high level of inhibition seen following exposure to quercetin metabolites, with platelets unable to activate fully due to inhibition of multiple key pathways.

Consistent with an effect on platelet aggregation, isorhamnetin and tamarixetin potently inhibited granule secretion, with an effect on α-granule secretion at submicromolar concentrations. The mechanisms underlying the inhibition of granule secretion were not investigated; however, flavonoids have been shown to interfere with SNARE (soluble NSF attachment protein receptor) complex formation in mast cells, and quercetin has also demonstrated actin-binding properties, inhibiting its polymerization. 37 38 In addition to this, inhibition of upstream activatory events (Ca ^2+^ mobilization, receptor-proximal signaling events, etc.) may suppress downstream granule secretion.

A significant inhibition of integrin αIIbβ3 function was also demonstrated. The initial conformational change opening the integrin into a high-affinity state that binds fibrinogen was strongly attenuated by quercetin, offering a mechanistic explanation for the observed reduction in aggregation. An increased potency of the aglycone over the methylated metabolites here may imply the presence of a potent uninvestigated metabolite. Indeed, quercetin is metabolized extensively upon consumption; Hong and Mitchell identified 17 metabolites in human urine following consumption of cooked onions, and Lee et al identified 15 metabolites in human plasma following enriched apple sauce consumption. 39 40 It may also be that, in vivo, it is the combinations of metabolites that act together to potently inhibit platelet function. In addition, whilst the methylated metabolites may demonstrate increased effects at lower flavonoid concentrations, quercetin may have a larger maximal effect on integrin inside-out signaling; the reason for this is unclear but may be due to structural differences in the flavonoids. The adhesion and spreading of platelets on a fibrinogen-coated surface was also reduced upon flavonoid treatment, with an effect on integrin outside-in signaling confirmed with the observation of reduced clot retraction upon flavonoid treatment. Thus, quercetin and its methylated metabolites dampen integrin αIIbβ3 function downstream of GPVI and PAR1/4 activation. Such inhibition of clot retraction may be mediated via specific antiplatelet effects, such as disruption of actin cytoskeletal arrangement and recruitment of cytoskeletal-associated proteins such as myosin heavy chain to β3. 41 In addition to this, Src family kinases (SFKs), specifically c-Src, have been shown to be crucial for integrin outside-in signaling, and quercetin potently inhibits SFKs including Fyn and Lyn. 35 42 An inhibition of platelet activation induced by thrombin here agrees with the GPCR-stimulated aggregometry presented in this study, and offers further evidence for quercetin and its metabolites as inhibitors of the thrombin-stimulated platelet activation pathway, even in the presence of plasma proteins. Quercetin has also exhibited anticoagulant activities in numerous studies, including inhibitory effects on the enzymatic activity of thrombin and Factor Xa. 43 44 Thus, the potential anticoagulant effects of quercetin's methylated metabolites must also be considered.

**Fig. 7:**
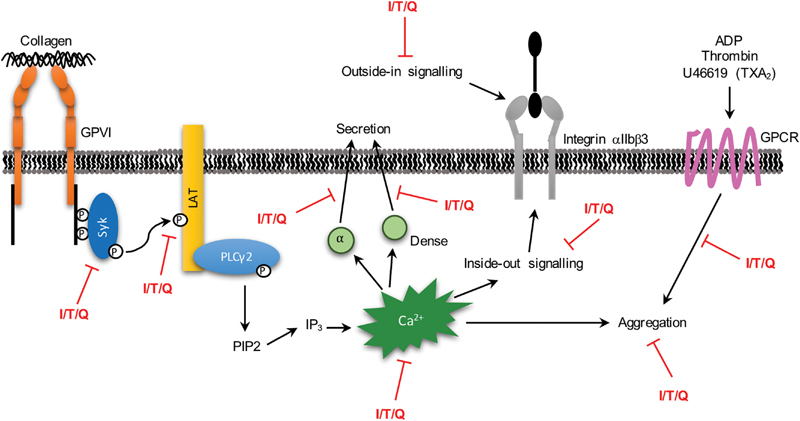
A model demonstrating the multiple platelet activation pathways inhibited by the methylated metabolites of quercetin. Isorhamnetin, tamarixetin, and quercetin inhibit multiple aspects of the GPVI activation pathway, including Syk and LAT phosphorylation, alpha and dense granule secretion, calcium mobilization, integrin αIIbβ3 inside-out signaling and, ultimately, aggregation (indicated by the *red text* ). In addition, this study demonstrates an inhibition of platelet aggregation downstream of ADP, thrombin, and U46619 stimulation. This multitude of inhibitory effects culminates in the potent inhibition observed upon treatment with isorhamnetin, tamarixetin, or quercetin, and results in attenuated thrombus formation. ADP, adenosine phosphate; GPCR, G-protein coupled receptor; GPVI, glycoprotein VI; I, isorhamnetin; IP3, inositol 1,4,5-trisphosphate; LAT, linker for activation of T cells; PLCγ2, phospholipase Cγ2; PIP2, phosphatidylinositol 4,5-bisphosphate; Q, quercetin; Syk, spleen tyrosine kinase; T, tamarixetin; TXA2, thromboxane A _2_ .

Many flavonoids have been shown to inhibit intracellular enzymes key in signal transduction. For example, in platelets alone, it has been demonstrated that citrus flavonoids such as nobiletin inhibit PI3K-mediated signaling cascades, and genistein, luteolin, apigenin, and quercetin inhibit total tyrosine phosphorylation and extracellular signal-regulated kinase (ERK)1/2 activation upon U46619 stimulation. 18 45 As such, and due to the observed inhibition of early activation events such as granule secretion in this study, it was hypothesized that signaling events proximal to the GPVI receptor may be attenuated by the methylated metabolites of quercetin. Indeed, the phosphorylation of Syk and LAT, very early signaling events in platelets upon GPVI activation, were inhibited potently by isorhamnetin, tamarixetin, and quercetin. This likely explains previous observations that collagen-stimulated phosphorylation of phospholipase Cγ2 is inhibited ex vivo after quercetin ingestion, as well as by quercetin and tamarixetin in vitro. 12 46 Consistent with this, CRP-XL-stimulated mobilization of calcium was reduced upon flavonoid treatment. This alteration of early platelet signaling events likely explains the potent functional inhibition observed, with the mobilization of calcium driving key processes such as integrin activation, TXA2 production, and granule secretion. 47 Indeed, inhibition early in the GPVI signaling pathway at the level of Syk and LAT (and subsequent calcium mobilization) is also likely to explain the inhibition of CRP-XL/collagen-stimulated granule secretion, fibrinogen binding, and platelet aggregation observed in this study, with these signaling molecules crucial to downstream platelet activation. The multitude of pathways and platelet functions inhibited by quercetin and its methylated metabolites in this study are displayed in Fig. 7 . In addition to this, a recent study by Doucet et al identified a peracetylated quercetin as a selective inhibitor of the 12-lipoxygenase pathway, a pathway essential for platelet activation through the low-affinity receptor for IgG, FcγRIIa; it may therefore be that the methylated metabolites of quercetin dampen platelet activation through this mechanism. 48 49

This study also demonstrates an antithrombotic effect of the methylated metabolites of quercetin, measured in vitro as an inhibition of thrombus formation under arterial flow conditions. A concentration of 10 μM was used in this assay, corresponding to a physiologically achievable total plasma quercetin (plus metabolite) concentration upon supplementation. 12 23 A trend toward increased potency of tamarixetin over quercetin and isorhamnetin was observed, similar to effects in clot retraction. These data therefore demonstrate the increased antiplatelet effect of a quercetin metabolite compared to the parent aglycone, offering insight into how quercetin consumption could lead to antiplatelet efficacy in vivo. The cause of increased potency is not established here, but there are several potential explanations for this. It has been demonstrated that tamarixetin binds collagen and fibrinogen with a much higher affinity than quercetin; this could interfere with platelet–ligand binding in this assay, which relies on a collagen-coated surface for platelet activaton. 46 A study by Dufour and Dangles observed a two to three times reduction in binding constants to bovine serum albumin upon methylation of quercetin at the 4′-hydroxyl residue; reduced binding to albumin may therefore result in greater levels of available metabolite and therefore an apparent increase in efficacy. 50 It is hypothesized that the enhanced potency of tamarixetin is not, therefore, due to increased antiplatelet activity per se, but rather altered bioavailability/platelet–ligand binding.

An antithrombotic effect was demonstrated in vivo upon oral supplementation in mice with 200 mg/kg isoquercetin over a 48-hour period. Thrombi were unstable and prone to embolization upon isoquercetin treatment; this may be due to impaired integrin αIIbβ3 outside-in signaling, which has a proven role in thrombus stabilization and is shown in the present study to be attenuated by quercetin and its methylated metabolites. 51 A recent study by Feng et al reported a similar phenotype in a knock-in mouse with impaired β3 phosphorylation which displayed delayed vessel occlusion and unstable thrombi upon injury. 52 Isoquercetin itself is unlikely to be identified in plasma after oral supplementation due to glycoside hydrolysis by enzymes such as enterocyte lactin-phlorizin hydrolase (LPH) and β-glucosidases. 53 Indeed, upon oral isoquercetin administration, Paulke et al identified isorhamnetin/tamarixetin in rat plasma. 29 The identification of the active metabolite(s) in the in vivo assay presented in this study, and their pharmacokinetic parameters, is therefore a primary goal of further research. A previous study from Hubbard et al also identified isorhamnetin and tamarixetin in human plasma upon ingestion of quercetin-4′-O-β-D-glucoside, a glucosidated form of quercetin similar to isoquercetin. 12 Isorhamnetin and tamarixetin displayed similar pharmacokinetic properties to quercetin, accumulating with a *t*_max_ between 30 and 45 minutes. 12 Thus, with repeated dosing through diet or supplementation, it may be possible that these metabolites (and other as yet unidentified metabolites) may accumulate in plasma or within platelets and exert their antiplatelet activities as established in this study. Indeed, a study evaluating metabolite identification and pharmacokinetics post-isoquercetin supplementation in humans is another goal of further research. Nevertheless, these data demonstrate both the oral bioavailability of isoquercetin and a protective effect against thrombosis.

Aspirin is one of the most widely used antiplatelet agents worldwide. 54 Its use is associated with adverse events such as gastric bleeding, and whilst studies have shown that decreasing aspirin doses reduces adverse event numbers without a reduction in antiplatelet efficacy, even low-dose aspirin treatment is associated with an increased bleeding risk. 31 32 As such, the pharmacological implications of the effects of the methylated metabolites of quercetin with respect to the antiplatelet effects of aspirin are worthy of consideration. A preliminary investigation in this study demonstrated that isorhamnetin, tamarixetin, and quercetin all increased the antiaggregatory effects of aspirin in a synergistic, i.e., more-than-additive manner, lowering IC _50_ values by over an order of magnitude at higher concentrations. In addition to this, isorhamnetin, tamarixetin, and quercetin enhanced the effect of aspirin in a whole blood assay of platelet function (PFA-100) more-than-additively. The mechanism underlying this interaction is not established here but could be for several reasons. Several studies have demonstrated the ability of quercetin to directly inhibit COX-1 activity; indeed, Al-Fayez et al established an IC _50_ of ∼5 μM on purified COX-1. 55 56 In vivo metabolites of quercetin may therefore be able to inhibit COX-1 at physiologically relevant levels. This holds clear implications for thromboxane synthesis. Indeed, Guerrero et al showed that quercetin can inhibit the production of thromboxane B2 downstream of collagen and arachidonic acid stimulation of platelets. 57 Co-administration with flavonoids may therefore allow further lowering of aspirin doses to be achieved. It must also be considered that such interactions between the effects of dietary flavonoids and aspirin may act as interfering factors in the use of antiplatelet agents, potentially explaining variability in response to such agents. Further research is needed to elucidate the potentially helpful (enhanced antithrombotic effect) or harmful (compromised hemostasis) effects of flavonoid–antiplatelet medication interactions in vivo.

Our study has demonstrated that isorhamnetin and tamarixetin inhibit platelet function and thrombus formation through effects on early activatory processes including calcium mobilization, granule secretion, and integrin activation. These metabolites, as well as quercetin aglycone, synergize with aspirin to enhance antiplatelet efficacy. Combined with the in vivo antithrombotic effect of isoquercetin demonstrated here, this may offer insight into new CVD treatment avenues, and offer evidence into the link between flavonoid intake and reduced CVD risk.
