# Supplementary material for: The Metabolites of the Dietary Flavonoid Quercetin Possess Potent Antithrombotic Activity, and Interact with Aspirin to Enhance Antiplatelet Effects
Source: TH Open. 2019 Jul 30;3(3):e244–58. doi: 10.1055/s-0039-1694028 (PMC6667742; doi:10.1055/s-0039-1694028)
Supplement: Supplementary file 3 — Supplementary Material [file 10-1055-s-0039-1694028-s190030.pdf]

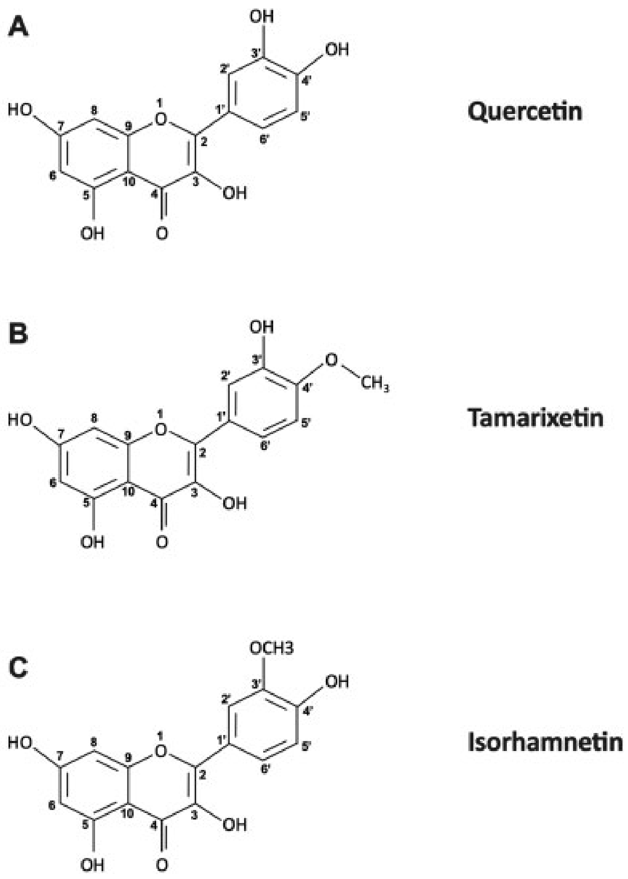

Supplementary Fig. S1 The structures of the compounds investigated: quercetin (**A**), tamarixetin (**B**), and isorhamnetin (**C**).

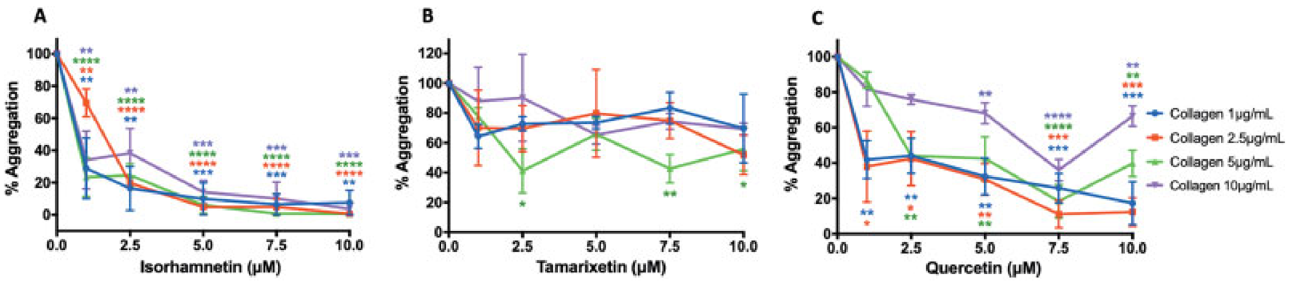

Supplementary Fig. S2 Quercetin and its methylated metabolites inhibit platelet aggregation evoked by multiple concentrations of collagen. Washed platelets (2×10^8 cells/mL) were incubated in a 96-well plate with isorhamnetin (**A**), tamarixetin (**B**), quercetin (**C**), or vehicle control (DMSO, 0.2% v/v) for 5 minutes, with aggregation subsequently measured via plate-based aggregometry. Data represent percentage aggregation with each agonist concentration normalized to levels of aggregation in the absence of flavonoid (vehicle). $N = 4$, data represent mean ± SEM. Data analyzed by one-way ANOVA with posthoc Dunnett's test, *colored stars* refer to statistical significance compared to vehicle control against the agonist indicated by the same color line in the legend. $^{*}p < 0.05$, $^{**}p < 0.005$, $^{***}p < 0.001$, $^{****}p < 0.0001$.

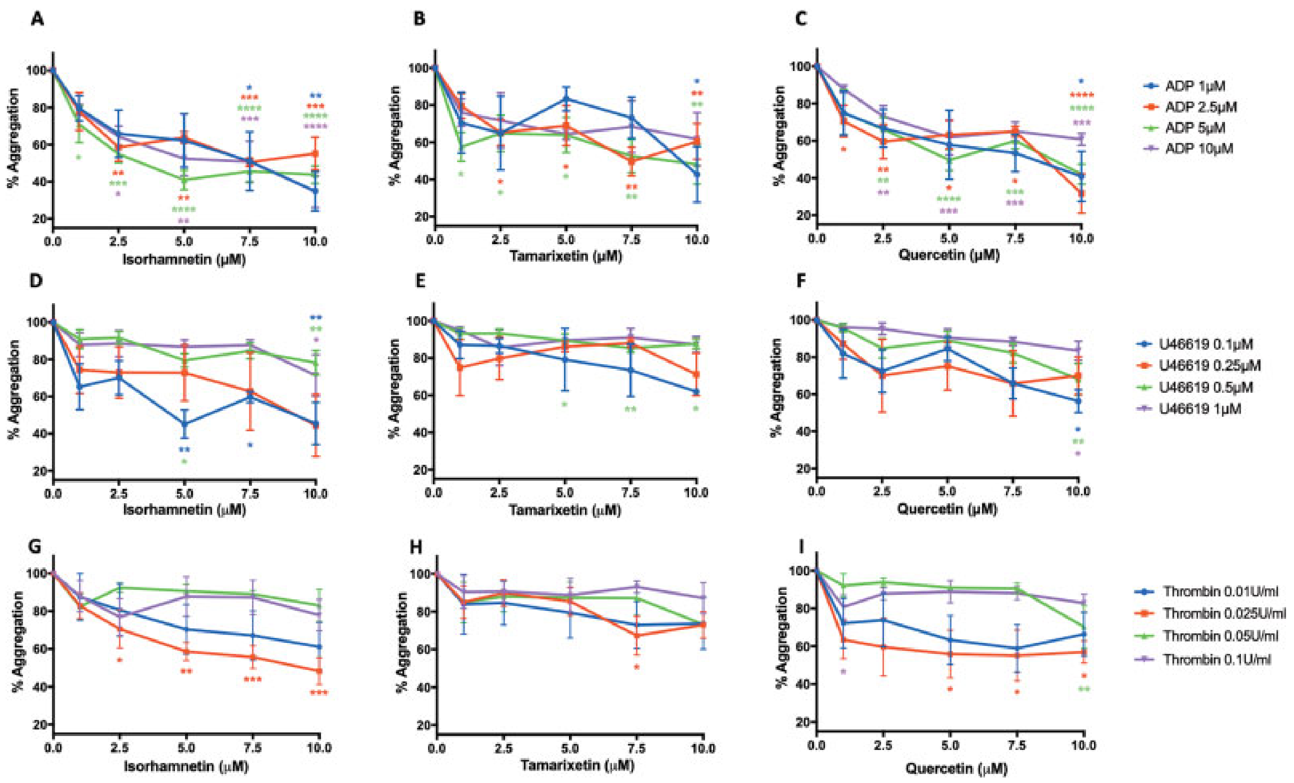

Supplementary Fig. S3 Isorhamnetin, tamarixetin, and quercetin inhibit platelet aggregation stimulated through G-protein coupled receptors. Washed platelets (2×10^8 cells/mL) were incubated in a 96-well plate with isorhamnetin (**A, D, G**), tamarixetin (**B, E, H**), quercetin (**C, F, I**), or vehicle control (DMSO, 0.2% v/v) for 5 minutes, with aggregation subsequently measured via plate-based aggregometry. Data represent percentage aggregation with each agonist concentration normalized to levels of aggregation in the absence of flavonoid (vehicle). $N = 4$, data represent mean ± SEM. Data analyzed by one-way ANOVA with posthoc Dunnett's test, *colored stars* refer to statistical significance compared to vehicle control against the agonist indicated by the same colur line in the legend. $^{*}p < 0.05$, $^{**}p < 0.005$, $^{***}p < 0.001$, $^{****}p < 0.0001$. ADP, adenosine diphosphate.

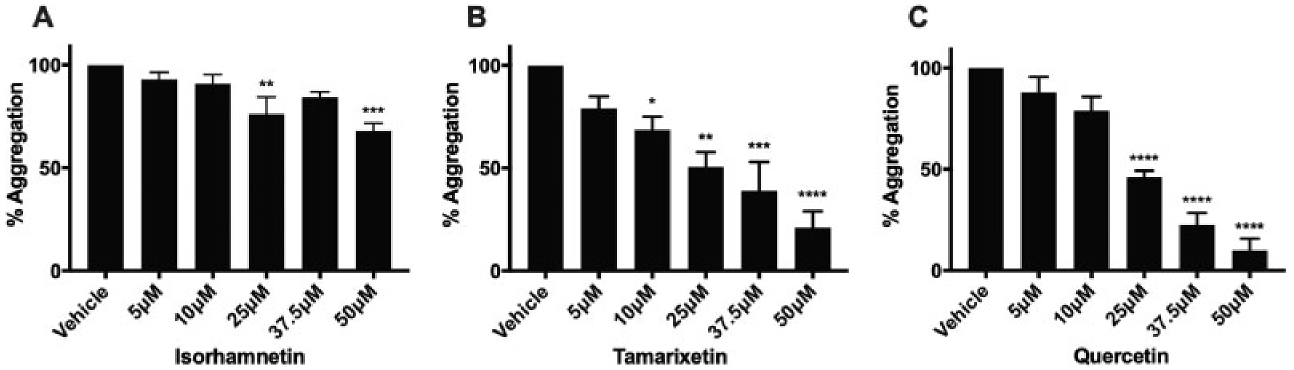

Supplementary Fig. S4 Isorhamnetin, tamarixetin, and quercetin inhibit platelet aggregation in platelet-rich plasma (PRP). PRP was incubated in a 96-well plate with isorhamnetin (**A**), tamarixetin (**B**), quercetin (**C**), or vehicle control (DMSO, 0.2% v/v) for 30 minutes at 37 °C prior to stimulation with collagen (5 µg/mL). Data represent percentage aggregation normalized to levels of aggregation in the absence of flavonoid (vehicle). $N = 4$, data represent mean ± SEM. $^{*}p < 0.05$, $^{**}p < 0.005$, $^{***}p < 0.001$, $^{****}p < 0.0001$, data analyzed by one-way ANOVA with posthoc Dunnett's test.
